# Notch signaling regulates the responses of lipopolysaccharide-stimulated macrophages in the presence of immune complexes

**DOI:** 10.1371/journal.pone.0198609

**Published:** 2018-06-11

**Authors:** Wipawee Wongchana, Pornrat Kongkavitoon, Pattarin Tangtanatakul, Chutamath Sittplangkoon, Patcharavadee Butta, Supatta Chawalitpong, Thitiporn Pattarakankul, Barbara A. Osborne, Tanapat Palaga

**Affiliations:** 1 Graduate Program in Biotechnology, Faculty of Science, Chulalongkorn University, Bangkok, Thailand; 2 Center of Excellence in Immunology and Immune-mediated Diseases, Chulalongkorn University, Bangkok, Thailand; 3 Department of Microbiology, Faculty of Science, Chulalongkorn University, Bangkok, Thailand; 4 Department of Veterinary and Animal Sciences, University of Massachusetts at Amherst, Amherst, Massachusetts, United States of America; 5 Omics Sciences & Bioinformatics Center, Chulalongkorn University, Bangkok, Thailand; Ohio State University, UNITED STATES

## Abstract

Macrophages exhibit diverse effector phenotypes depending on the stimuli and their microenvironment. Classically activated macrophages are primed with interferon (IFN)γ and stimulated with pathogen-associated molecular patterns. They produce inflammatory mediators and inflammatory cytokines, such as IL-12. In the presence of immune complexes (ICs), activated macrophages have decreased IL-12 production and increased IL-10 production and presumably act as regulatory macrophages. Notch signaling has been shown to regulate the effector functions of classically activated macrophages. In this study, we investigated whether Notch signaling is active in lipopolysaccharide (LPS)-stimulated macrophages in the presence of ICs. LPS/IC stimulation increased the level of cleaved Notch1 in murine macrophages, while IC stimulation alone did not. Delta-like 4, but not Jagged1, was responsible for generating cleaved Notch1. The activation of Notch signaling by LPS/ICs depended upon NF-κB and MEK/Erk pathway activation. Macrophages with the targeted deletion of *Rbpj*, which encodes a DNA-binding protein central to canonical Notch signaling, produced significantly less IL-10 upon LPS/IC stimulation. A similar impact on IL-10 production was observed when Notch signaling was inhibited with a gamma-secretase inhibitor (GSI). Defects in NF-κB p50 nuclear localization were observed in GSI-treated macrophages and in *Rbpj*^*-/-*^ macrophages, suggesting cross-regulation between the Notch and NF-κB pathways. Transcriptomic analysis revealed that Notch signaling regulates the transcription of genes involved in the cell cycle, macrophage activation, leukocyte migration and cytokine production in LPS/IC-stimulated macrophages. Taken together, these results suggest that the Notch signaling pathway plays an important role in regulating the functions of macrophages activated by LPS and ICs.

## Introduction

Macrophages mediate both innate and adaptive immune responses. Signaling through lipopolysaccharide (LPS)/TLR4 results in the execution of host defense functions, such as phagocytosis and killing activities, by macrophages [1], and the cascade of downstream signaling molecules that are induced by LPS facilitates the transcriptional activation of inflammatory-associated cytokines, such as TNFα, IL-1β, IL-6, IL-12, and type I interferon, as well as the production of relatively low amounts of IL-10. Additionally, the priming of macrophages with IFNγ enhances TLR-induced cytokine gene expression, partly by facilitating the remodeling of chromatin to increase chromatin accessibility and the recruitment of TLR-induced transcription factors to the regulatory promoter regions [2]. These macrophages are well-characterized as classically activated macrophages [3].

Alternatively, macrophages can be activated by signaling through Fc gamma receptor (FcγRs) via antigen-antibody complexes. Immune complexes (ICs) and IgG-opsonized pathogens or particles bind to FcγRs expressed on the surfaces of macrophages; FcγRs are functionally characterized as activation or inhibitory receptors [4]. Mosser *et al*. reported a unique phenotype of activated macrophages that are stimulated by IFNγ/LPS in the presence of ICs. This stimulation leads to macrophage activation that yields high levels of IL-10 and low levels of IL-12 while maintaining the levels of other innate cytokines, such as TNFα. In addition to these signature cytokines, LPS/IC-activated macrophages also express unique gene expression profiles that are different from classically activated macrophages or IL-4-stimulated macrophages, the so-called M2 macrophages [5, 6]. Because of the cytokine profiles opposite those of classically activated macrophages, at least those for IL-12 and IL-10, these macrophages are considered to be distinct from classically activated macrophages and are called type II or regulatory (also called M2b or M(IC)) macrophages [3, 7, 8]. The adoptive transfer of these regulatory macrophages alleviates the severity of autoimmune disease in a mouse model of experimental autoimmune encephalomyelitis (EAE), suggesting that they have a systemic impact *in vivo* [9].

IL-10 is one of the key signature cytokines of LPS/IC-activated macrophages; IL-10 causes these macrophages to function as regulatory cells during the immune activation state. The role of IL-10 produced by IC-stimulated macrophages is indicated by the worsening outcomes of some infectious diseases caused by intracellular pathogens [10]. Furthermore, macrophages activated by TLR ligands in the presence of ICs are linked to some autoimmune diseases, particularly systemic lupus erythematosus (SLE) and rheumatoid arthritis (RA) [11, 12]. Because IL-10 functions as a regulatory cytokine that is important for controlling the inflammatory process, the regulatory mechanism of IL-10 expression has been extensively studied in immune cells, including macrophages [13, 14]. In macrophages, the transcription of *Il10* mRNA is selectively regulated by various transcription factors, including Erk, Sp1 and NF-κB. The production of IL-10 is induced in TLR-dependent and TLR-independent manners in macrophages. In LPS-activated macrophages, IL-10 is produced at relatively low levels, and its transcription is controlled mainly by the NF- κB pathway (p50 and p65) and the MAPK and STAT pathways [15–17]. Signaling through FcγRs in LPS/IC-stimulated macrophages amplifies the activation of Erk and p38 MAPK signaling, thus augmenting chromatin remodeling and the binding of Sp1 to the *Il10* promoter [18]. Furthermore, PI3K/AKT signaling downstream of FcγRs is also responsible for optimal IL-10 expression [19]. Although detailed signaling pathways involving TLRs and FcγRs have been reported in the regulation of IL-10 production, the involvement of other signaling pathways, including Notch signaling, remains largely unexplored.

The Notch signaling pathway regulates multiple cellular processes, including differentiation, proliferation and survival [20]. Notch signaling comprises four Notch receptors (Notch1-4), five ligands (Delta-like (Dll) 1, 3 & 4 and Jagged 1 & 2) and the DNA binding protein CSL/RBP-Jκ. The interactions between Notch ligands and receptors induce the sequential enzymatic cleavage of Notch receptors by ADAM metalloprotease and gamma-secretase, resulting in the release of the intracellular domain of the Notch receptor. The cleaved Notch receptor forms a complex with CSL/RBP-Jκ in the nuclei, and together, they regulate the transcription of Notch target genes [21]. We and others demonstrated that TLR-activated macrophages induced the expression of the full-length Notch1 receptor as well as the production of cleaved Notch receptors [22, 23]. Signaling downstream of TLRs induces expression of Jagged1 in NF-κB and MAPK dependent manner. Jagged1/Notch create an autoamplification loop of Notch signaling that can be enhanced by IFNγ [24]. TLR and Notch together induces expression of the Notch target genes, *Hes1* and *Hey1*. These two proteins attenuate the expression of IL-6 and IL-12, the effect that can be surpassed by IFNγ treatment [23]. The activation of Notch signaling through TLR activation is important for the production of pro-inflammatory cytokines, including TNFα, IL-6, IL-10 and IL-12, in TLR-activated macrophages [22, 25, 26]. Thus, Notch signaling plays important roles in the polarization and activation of pro-inflammatory macrophages [23, 26–29].

There are reports that Notch signaling regulates IL-10 production in other immune cells. For example, Notch signaling promotes *Il10* mRNA expression via STAT4 in Th1 cells [30]. Previously, our group demonstrated that Notch signaling affects the activation of NF-κB p50 and p65 in LPS-activated macrophages, implying that Notch signaling may be involved in the production of cytokines that are targets of NF-κB signaling [22]. However, whether Notch signaling is active in LPS/IC-stimulated macrophages and/or plays a role in regulating cellular functions, such as IL-10 production, have not been elucidated. Here, we showed that in LPS/IC-stimulated macrophages, Notch signaling is activated, and the crosstalk among Notch signaling, signaling downstream of FcγRs and TLR signaling cooperates to regulate gene expression, including IL-10, in LPS/IC-stimulated macrophages. This work highlights the complex role that Notch signaling plays in inflammatory and regulatory macrophages.

## Materials and methods

### Animals

Wild type C57BL/6 mice were purchased from the National Laboratory Animal Center (Salaya, Thailand). Conditional *Rbpj* KO mice were generated as described previously [31]. All animal procedures were approved by the Institutional Animal Care and Use Committees (IACUCs) of Chulalongkorn University and the University of Massachusetts at Amherst and performed according to the guidelines approved by the IACUCs (Protocol Review No. 1323007). Mice were humanely sacrificed by CO_2_ inhalation in the euthanasia chamber.

### Generation of bone marrow-derived macrophages (BMMs)

Bone marrow cells were flushed from the femur cavities of mice and used for generating BMMs as described elsewhere [22]. In brief, 5 x 10^6^ bone marrow cells were plated in non-tissue culture treated plates (Hycon, Thailand) and cultured at 37°C and 5% CO_2_ in DMEM supplemented with 10% fetal bovine serum, HEPES, sodium pyruvate, streptomycin/penicillin G (all from LONZA, USA, or HyClone, UK), 5% (v/v) horse serum (Thermo Scientific, USA) and 20% (v/v) L929-conditioned media. Fresh DMEM supplemented with 20% L929-conditioned media and 5% horse serum was added to the cultures on day 4. The cells were harvested on day 7 and used for all the experiments.

### Activation of BMMs

BMMs were primed overnight with recombinant murine IFNγ (10 ng/mL) (BioLegend, USA) and washed twice with media and warm PBS. *Salmonella* LPS (100 ng/mL) (Sigma-Aldrich, USA), purified rabbit IgG against ovalbumin (OVA) (GeneTex, USA) and IgG-opsonized OVA (immune complex) were added to activate the macrophages as indicated. GSI, DAPT (25 μM) (Calbiochem, USA) and vehicle control DMSO (0.01%) (Sigma-Aldrich, USA) were incubated with the macrophages overnight before activation. BAY-11(10 μM), SB203580 (10 μM), U0126 (10μM), Y294002 (50 μM) (all inhibitors were purchased from Calbiochem, USA) or DMSO (0.01%) (Sigma-Aldrich, USA) was used to pretreat the macrophages for 30 min before activation. Macrophage activation was confirmed by measuring *Il12b* and *Il10* mRNA expression by quantitative RT-PCR (qPCR).

### Preparation of the ICs

The ICs were prepared as described previously [5]. Briefly, a 10-fold molar excess of purified rabbit anti-OVA IgG (GeneTex, USA) or rabbit anti-OVA IgG (Sigma-Aldrich, USA) was mixed with OVA (Sigma-Aldrich, USA) and incubated for 30 min at room temperature. To activate the macrophages, a 1:100 volume ratio of the immune complexes to media was used for culture, in addition to LPS.

### Intracellular staining and cell surface staining

For intracellular staining, monensin was added at the start of the activation. Fc receptors were blocked with a FACs staining buffer containing Fc blocker (0.5 μg) (BD Bioscience, USA), followed by cell surface staining and fixation/permeabilization using a BD Cytofix/Cytoperm kit (BD Biosciences, USA) according to the manufacturer’s instructions. For IL-10, an anti-mouse IL-10-PE antibody (0.4 μg) (BioLegend, USA) was used. For detecting Notch ligands, anti-hamster Jagged1-PE, anti-hamster Jagged2-PE, anti-hamster Dll1-PE and anti-hamster Dll4-PE (BioLegend, USA) were used for cell surface staining. The cells were sorted on a Cytomics FC 500 MPL cytometer (Beckman Coulter, USA) and analyzed using FlowJo software (Tree Star, CA, USA).

### Preparation of cytosolic and nuclear extracts

BMMs (wildtype or *Rbpj* KO mice) were activated and treated as indicated. Cytosolic and nuclear extracts were prepared using NE-PER^™^ Nuclear and Cytoplasmic Extraction Reagents according to the manufacturer’s instruction (Thermo Fisher Scientific, USA). Protein concentrations were measured using Pierce^™^ BCA Protein Assay Kit (Thermo Fisher Scientific, USA). Equal amount of proteins were analyzed by Western blots.

### Blocking of Notch ligands

BMMs were primed with IFNγ overnight in the presence of Jagged 1-specific blocking antibody (BioLegend, USA) or Dll4-specific blocking antibody (BioXcell, USA) or isotype controls. After washing in PBS, cells were stimulated with LPS and OVA-IgG IC as indicated above. Cell lysates were analyzed by Western blot and total RNA was extracted and used for determine mRNA expression.

### Western blots

BMMs were activated as indicated, and the protein lysates were subjected to Western blots. The primary antibodies used in this study were as follows: rabbit anti-Notch1 (1:2000) (Santa Cruz Biotechnology, USA), rabbit anti-cleaved Notch1 (Val1744) (1:1000), rabbit anti-phospho-p38 (1:2000), rabbit anti p38 (1:2000), rabbit anti-phospho-p44-42 (1:4000), rabbit anti p44-42 (1:4000), rabbit anti-phospho-SAPK-JNK (1:2000), rabbit anti-SAPK-JNK (1:2000), rabbit anti-phospho-AKT (1:2000), rabbit anti-AKT (1:2000) and rabbit anti-RBPJSHU (1:1000) (all from Cell Signaling Technology, USA), mouse anti β-actin (1:1000) (Chemicon-Millipore, USA) and rabbit anti-GAPDH (1:4000) (Santa Cruz Biotechnology, USA). The secondary reagents conjugated with horse-radish peroxidase (HRP) were as follows: donkey anti-rabbit IgG (1:2000–1:4000) and sheep anti-mouse IgG (1:5000) (Amersham Biosciences, UK). The signals were detected by chemiluminescence on X-ray films.

### RNA extraction and qRT-PCR

BMMs were activated as indicated, and total RNA was isolated by using TriZol reagent (Invitrogen, UK) or an RNeasy Mini Kit (Qiagen, Germany). cDNA was synthesized, and the transcripts were amplified by using a Mini-Opticon or CFX Connect^™^ real-time PCR detection system (Bio-Rad, USA). The primer sequences used are provided in S1 Table. The expression of each gene was normalized to the expression of β-actin by the 2^-ΔΔCT^ method.

### ELISA

Culture supernatants from BMMs treated as indicated were harvested at the indicated times after stimulation. Secreted IL-12p70 and IL-10 levels were detected by using an IL-12p40/70 ELISA (BD Pharmingen, USA) and IL-10 ELISA (BioLegend, USA). ELISAs was performed according to the manufacturer’s instructions.

### Immunofluorescent staining

Cells were cultured in an 8-well slide chamber and activated as indicated. After washing with PBS, the cells were fixed in 4% paraformaldehyde and blocked with Fc blocker (0.5 μg). The cells were washed and incubated with an anti-NF-κB p50 monoclonal antibody (1:100) (Santa Cruz Biotechnology, USA) and then an anti-mouse IgG (H+L) (Fab)_2_ fragment)-conjugated Alexa Fluor^®^ 488 (Cell Signaling Technology, MA, USA) (1:500) secondary antibody. Nuclei were stained by DAPI (Cell Signaling Technology, USA). The cells were observed under an inverted fluorescence microscope or a confocal microscope (Olympus, Japan).

### RNA sequencing and data analysis

Total RNA samples were extracted from LPS/IC-activated BMMs in the presence of DMSO or GSI for 1 hr using an RNeasy Mini Kit (Qiagen, USA). RNA samples were assessed for quality using an Agilent 2100 Bioanalyzer (Agilent, USA) and quantified using a Qubit 3.0 fluorometer (Life Technologies, USA). Strand-specific cDNA library preparation was performed using a TruSeq stranded mRNA library prep kit (Illumina, USA). The cDNAs were sequenced on a NextSeq 500 system (Illumina, USA) at the Omics Sciences and Bioinformatics Center (Chulalongkorn University). The obtained data were trimmed of Illumina adapters using Trimmomatic and aligned using STAR software. Raw reads were counted using HTSeq, and the reads were mapped against the mm10 reference genome and annotated with Entrez Gene. Differential gene expression (DE) was determined using edgeR software, and the statistics were calculated according to a quasi-negative binomial distribution. Statistical significance was indicated by an FDR cut-off of < 0.05. Heat-maps were generated by MultiExperiment Viewer (MeV 4.9). Functional annotations were performed using ToppGene Suite (http://toppgene.cchmc.org). Data has been deposited in NCBI Gene Expression Omnibus (GEO) and is accessible through GEO accession number GSE114020.

### Statistical analysis

Statistical analyses were performed using SPSS version 15.0 and GraphPad Prism version 5.0. Student’s t-test (paired or unpaired) and one-way ANOVA (α = 0.05) with Tukey’s multiple comparison test were used when comparing different conditions.

## Results

### Expression of IL-10 and IL-12p40 in LPS/IC-activated macrophages

**Fig 1:**
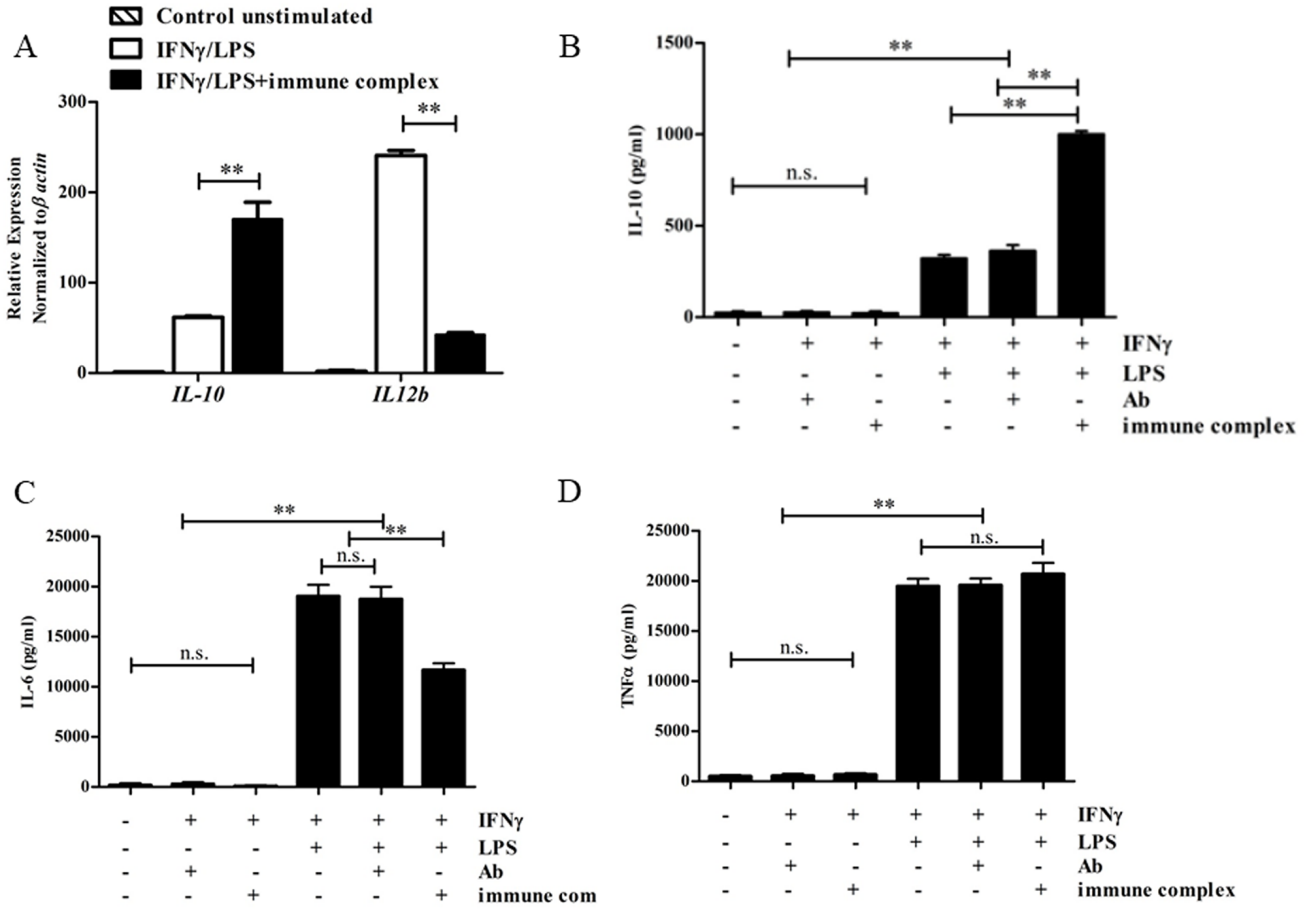
Cytokine expression in macrophages stimulated with LPS or LPS/ICs. (A) IFNγ-primed BMMs were activated by LPS (100 ng/mL) or LPS/ICs for 4 hrs. The expression levels of *Il10* and *Il12b* mRNA were measured by qPCR. (B-D) IFNγ-primed BMMs were activated by ICs (anti-OVA IgG) in the presence or absence of LPS (100 ng/mL) for 6 hrs. The levels of IL-10 (B), IL-6 (C) and TNFα (D) in the culture supernatant were measured by ELISA. ** indicates statistical significance (*p*<0.05) according to one-way ANOVA with Tukey’s multiple comparison test. n.s. indicates no statistical significance. The results represent the mean±SD of triplicate determinations from one representative experiment of two independent experiments.

To confirm the cytokine profiles in macrophages activated by LPS or LPS with ICs, the relative levels of *Il10* and *Il12b* mRNA were determined 4 hrs after activation. Consistent with previous reports, compared with those activated by LPS, macrophages activated by LPS and ICs showed significantly higher levels of *Il10* mRNA, whereas the levels of *Il12b* mRNA were significantly lower (Fig 1A) [5]. To determine the effect of IC treatment on cytokine production, IL-10 levels were measured in macrophages activated with various stimuli. As shown in Fig 1B, IL-10 production was detected in all LPS-stimulated conditions, while priming with IFNγ alone or ICs alone did not result in detectable IL-10 levels. As expected, compared to that stimulated by other conditions, IL-10 production reached the highest level upon activation by LPS and ICs (Fig 1B). Furthermore, stimulation with LPS in the presence of free antibody alone did not increase the amount of IL-10, suggesting that IC crosslinking is essential for enhancing IL-10 production.

We next measured the levels of pro-inflammatory cytokines, *i*.*e*., TNFα and IL-6, in LPS-stimulated macrophages the presence of ICs [12, 32, 33]. We found that TNFα was produced at comparable levels in the presence or absence of ICs, whereas the production of IL-6 was dampened in the presence of ICs, compared with that in the presence of LPS alone (Fig 1C and 1D). These data are consistent with previous reports and indicate that the crosslinking of FcγRs by ICs is essential for tipping the balance of cytokine production, which is accomplished mainly by decreasing IL-6 and IL-12 levels and increasing IL-10 levels [5].

### Activation of Notch signaling in LPS/IC-activated macrophages

**Fig 2:**
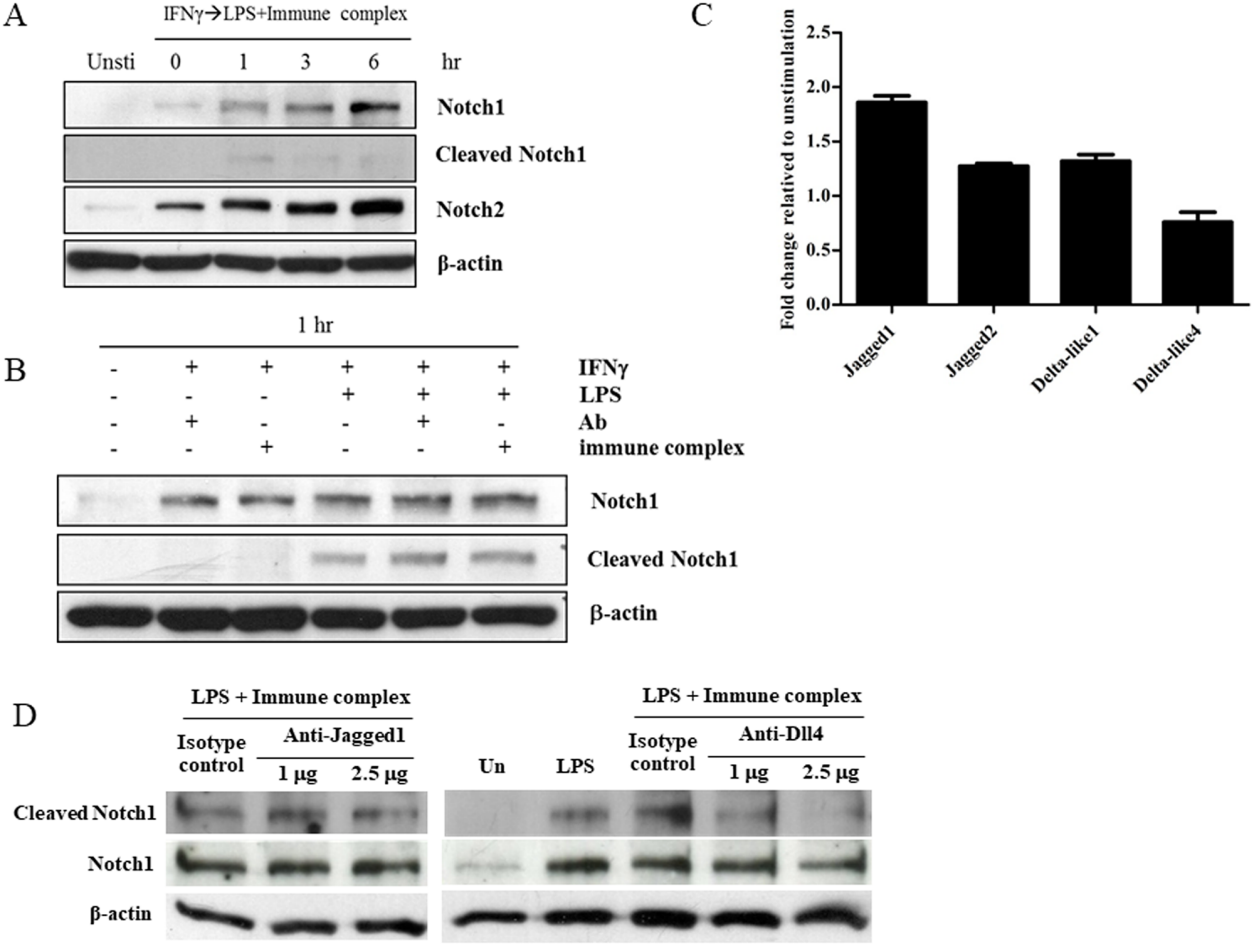
Stimulation by LPS is responsible for the activation of Notch signaling in LPS/IC-activated macrophages. (A) IFNγ-primed BMMs were activated by LPS/ICs for 0, 1, 3 and 6 hrs. Notch1, Notch2 and cleaved Notch1 (Val1744) were detected in whole cell lysates by Western blotting. β-actin was used for the loading control. Representative data from 1 of 2 independent experiments are shown. (B) BMMs were activated by anti-OVA IgG or ICs in the presence or absence of LPS (100 ng/mL) for 1 hr. Notch1 and cleaved Notch1 (Val 1744) were detected by Western blotting. β-actin was used as the loading control. Representative data from 1 of 2 independent experiments are shown. (C) Expression levels of Jagged1, Jagged2, Dll1 and Dll4 were detected by flow cytometry, and the relative levels were normalized to those from the unstimulated conditions. The results represent the mean±SD of triplicate determinations from one representative experiment of two independent experiments. (D) Cell lysates prepared from anti-Jagged1, anti-Dll4 antibody blocking or isotype control were analyzed for Notch1 and cleaved Notch1 (Val 1744) by Western blotting. Representative data from one of two independent experiments are shown. β-actin was used as the loading control.

Next, we determined whether Notch receptors and their ligands are expressed and whether the signaling is activated in LPS/IC-activated macrophages. First, the presence of cleaved Notch1 as an indicator of active Notch signaling was determined. The appearance of cleaved Notch1 was readily detectable at 1 hr after stimulation and persisted for at least 6 hrs. In parallel, the levels of Notch1 and 2 were also upregulated (Fig 2A). To investigate the stimuli contributing to the activation of Notch signaling in this setting, the cells were activated by LPS, anti-OVA antibody alone, ICs alone or the combination of LPS and ICs or anti-OVA antibody. As shown in Fig 2B, the activation of Notch signaling, as determined by the appearance of cleaved Notch1, was detected in only the condition where LPS was used, while anti-OVA antibody alone or ICs alone did not result in the appearance of cleaved Notch1. Therefore, signaling through TLR4 is crucial for the activation of Notch signaling. At 3 hrs after activation, higher levels of surface expression of the Notch ligand Jagged1 were observed compared to the other ligands while the level of Dll4 decreased (Fig 2C). Therefore, macrophages activated by LPS/ICs had upregulated expression levels of Notch1 and Notch2 and at least one Notch ligand. More importantly, the activation of Notch signaling was initiated after stimulation with LPS/ICs. These data indicated that the cleavage of Notch1 in LPS/IC-activated macrophages depends solely upon TLR4 signaling induced by LPS stimulation.

To investigate which Notch ligand(s) is responsible for Notch1 cleavage in LPS/IC-activated macrophages, specific antibody blocking was performed. As shown in Fig 2D, blocking Jagged1 did not show any effect on the level of cleaved Notch1 while blocking Dll4 clearly reduced the level of cleaved Notch1. These results indicated that Dll4 is likely the ligand responsible for Notch1 activation in LPS/IC-activated macrophages.

### LPS/IC stimulation initiated the activation of Notch signaling in a NF-κB and MAPK-dependent manner

**Fig 3:**
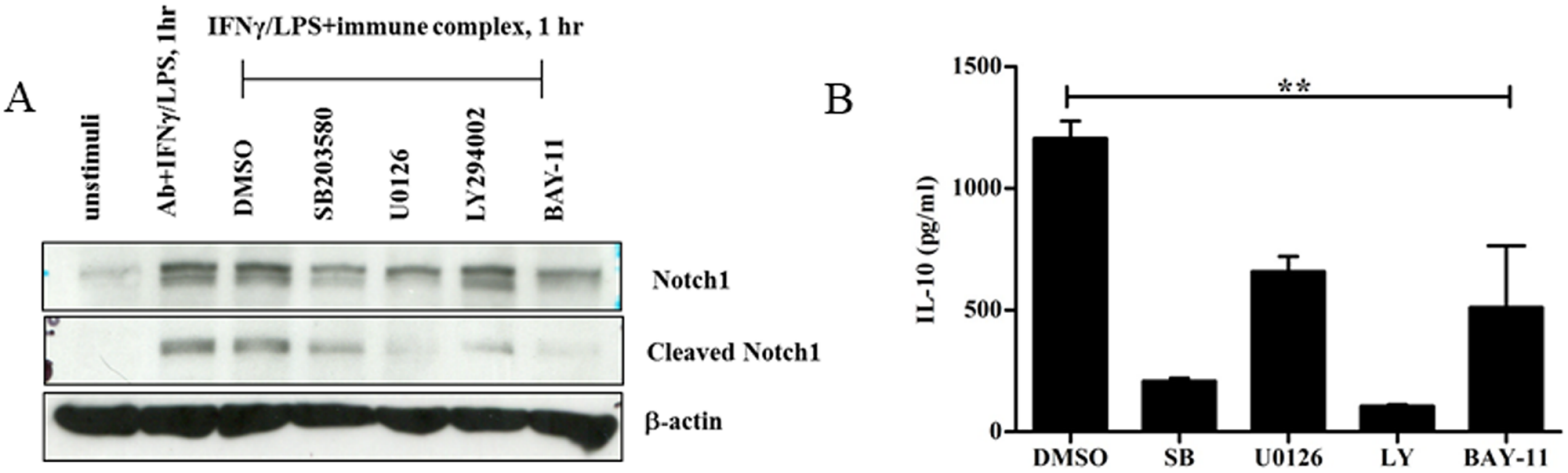
Effect of specific inhibitors of the MAPK, NF-κB and PI3K signaling pathways on Notch signaling activation and IL-10 production in LPS/IC-activated macrophages. (A) IFNγ-primed BMMs were pre-treated with Bay-11 (10 μM), SB203580 (10 μM), U0126 (10 μM), LY94002 (50 μM) or vehicle control DMSO for 30 min. The cells were subsequently activated by LPS/ICs for 1 hr, and protein lysates were created. Notch1 and cleaved Notch1 (Val 1744) were detected by Western blot. β-actin was used as the loading control. Representative data from one representative experiment of 3 independent experiments are shown. (B) Culture supernatants from the cells treated as in (A) were collected 6 hrs after stimulation, and the IL-10 levels were measured by ELISA. ** indicates statistical significance (*p*<0.05) according to one-way ANOVA with Tukey’s multiple comparison test. The results represent the mean±SD of triplicate determinations from one representative experiment of 2 independent experiments.

Foldi *et al*. reported that the activation of Notch signaling in TLR agonist-stimulated macrophages was initiated by Jagged1 auto-amplification through the NF-κB and MAPK signaling pathways [24]. Therefore, we asked whether the activation of Notch signaling, as determined by the presence of cleaved Notch1, with LPS/ICs is dependent on the NF-κB and/or MAPK pathways, similar to what was observed in LPS-activated macrophages. We used pharmacological inhibitors to specifically inhibit the NF-κB pathway (Bay-11), Erk/MEK1/2 pathway (U0126), p38 MAPK pathway (SB203580) and PI3K/Akt pathway (LY94002) and detected the appearance of cleaved Notch1. As shown in Fig 3A, cleaved Notch1 almost completely disappeared after macrophages were pretreated with either an NF-κB inhibitor or an Erk inhibitor, whereas Notch1 activation was only partially reduced after pretreatment with a p38 MAPK inhibitor or a PI3K inhibitor (Fig 3A). Therefore, the activation of Notch signaling was NF-κB-dependent and/or Erk/MEK-dependent in LPS/IC-activated macrophages, while the requirement of p38 MAPK and PI3K for Notch signaling was minimal.

To confirm the effect of these inhibitors on IL-10 production in LPS/IC-activated macrophages, IL-10 levels were quantitated by ELISA. As shown in Fig 3B, all inhibitors affected the expression of IL-10 to varying degrees. Treatment with specific inhibitors of p38 MAPK and PI3K resulted in a profound reduction in IL-10, whereas inhibitors of the Erk/MEK1/2 and NF-κB pathways resulted in partial reductions in IL-10; these findings indicate that p38 and PI3K are the main signaling pathways regulating IL-10 production, while the Erk/MEK1/2 and NF-κB pathways partially contribute to this regulation (Fig 3B). Taken together, the NF-κB and Erk/MEK pathways play an important role in the activation of Notch signaling and partially regulate IL-10 production in LPS/IC-activated macrophages.

### Effect of GSI treatment and CSL/RBP-Jκ deletion on IL-10 production in LPS/IC-activated macrophages

**Fig 4:**
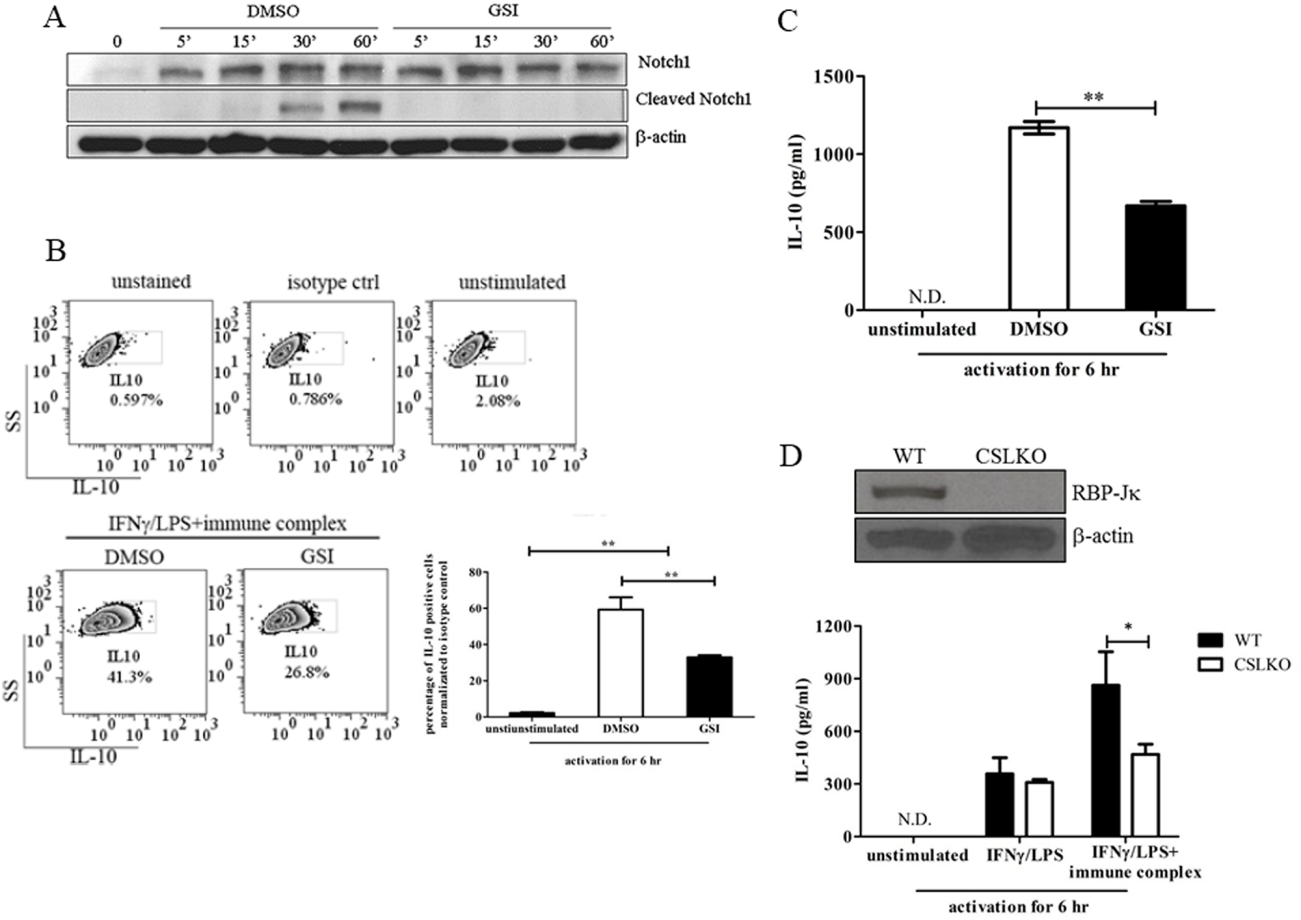
Effects of inhibiting Notch signaling by GSI or targeted *Rbpj* deletion on IL-10 production in LPS/IC-activated macrophages. (A) IFNγ-primed BMMs were pre-treated with vehicle control DMSO or GSI (25 μM) for 30 min. The cells were subsequently activated by LPS/ICs for 5, 15, 30 and 60 min. Cleaved Notch1 (Val 1744) and Notch1 were measured in whole cell lysates by Western blotting. Representative data from one of two independent experiments are shown. (B) IFNγ-primed BMMs were pre-treated with vehicle control DMSO or GSI (25 μM) and subsequently activated by LPS/ICs for 6 hrs. IL-10 expression was detected by intracellular cytokine staining. Representative data from one of two independent experiments are shown. (C) Culture supernatants were collected from BMMs stimulated for 6 hrs as described in (B), and the IL-10 levels were measured by ELISA. (D) CSL/RBP-Jκ expression in BMMs from wildtype (ctrl) or *Rbpj* KO (CSL KO) mice was detected by Western blotting. Representative data from one of two independent experiments are shown. BMMs from wildtype (ctrl) or *Rbpj* KO mice were activated by LPS or LPS/ICs for 6 hrs. IL-10 levels were detected by ELISA. * and ** indicate statistical significance (*p*<0.05) according to one-way ANOVA with Tukey’s multiple comparison test. The results represent the mean±SD of triplicate determinations from one of two independent experiments.

To study the role that Notch signaling plays in regulating IL-10 production, GSI was used to inhibit the cleavage of Notch receptors. The appearance of cleaved Notch1 was examined to validate the efficacy of GSI in inhibiting Notch signaling activation. Cleaved Notch1 was not produced upon GSI treatment, indicating that GSI treatment effectively inhibited the activation of the Notch signaling pathway (Fig 4A). Furthermore, the level of *hes1*, one of Notch target genes, was significantly reduced (S1A Fig). The percentage of cells producing IL-10 in LPS/IC-stimulated macrophages decreased by almost 50% for GSI-treated cells, compared with the mock control-treated cells, as measured by intracellular IL-10 staining (Fig 4B). This reduction at the cellular level reflected the *Il10* mRNA level and secreted IL-10 in the supernatant (S1B Fig and Fig 4C). GSI treatment also reduced the level of IL-10 in LPS-activated macrophages but to a lesser extent than that observed in LPS/IC-stimulated macrophages. Interestingly, the percentages of cells expressing IL-10 in LPS/IC-activated macrophages in the presence of GSI was comparable to that of IFNγ/LPS-activated macrophages (data not shown).

To confirm the impact of canonical Notch signaling on IL-10 production, bone marrow-derived macrophages from mice with targeted *Rbpj* deletion were used (Fig 4D). The level of IL-10 was significantly reduced in *Rbpj* knockout (KO) macrophages that were activated by LPS/ICs compared with that in the wildtype control macrophages (Fig 4D). However, IL-10 production was not decreased in *Rbpj* KO LPS-activated macrophages, in contrast to the findings observed for GSI treatment. The effect of Dll4 blocking on *Il10* expression was examined and the results show that blocking of Dll4, but not Jagged1, reduced the *i110* mRNA level in LPS/IC stimulated macrophages (S1C Fig. These data implied that the activation of canonical Notch signaling, which requires Dll4, the activity of gamma-secretase and the presence of CSL/RBP-jκ, is critical for IL-10 production in macrophages activated by LPS/ICs but not in macrophages activated by LPS alone.

### Effects of GSI treatment and deletion of CSL/RBP-Jκ on signaling downstream of TLR and FcγR

**Fig 5:**
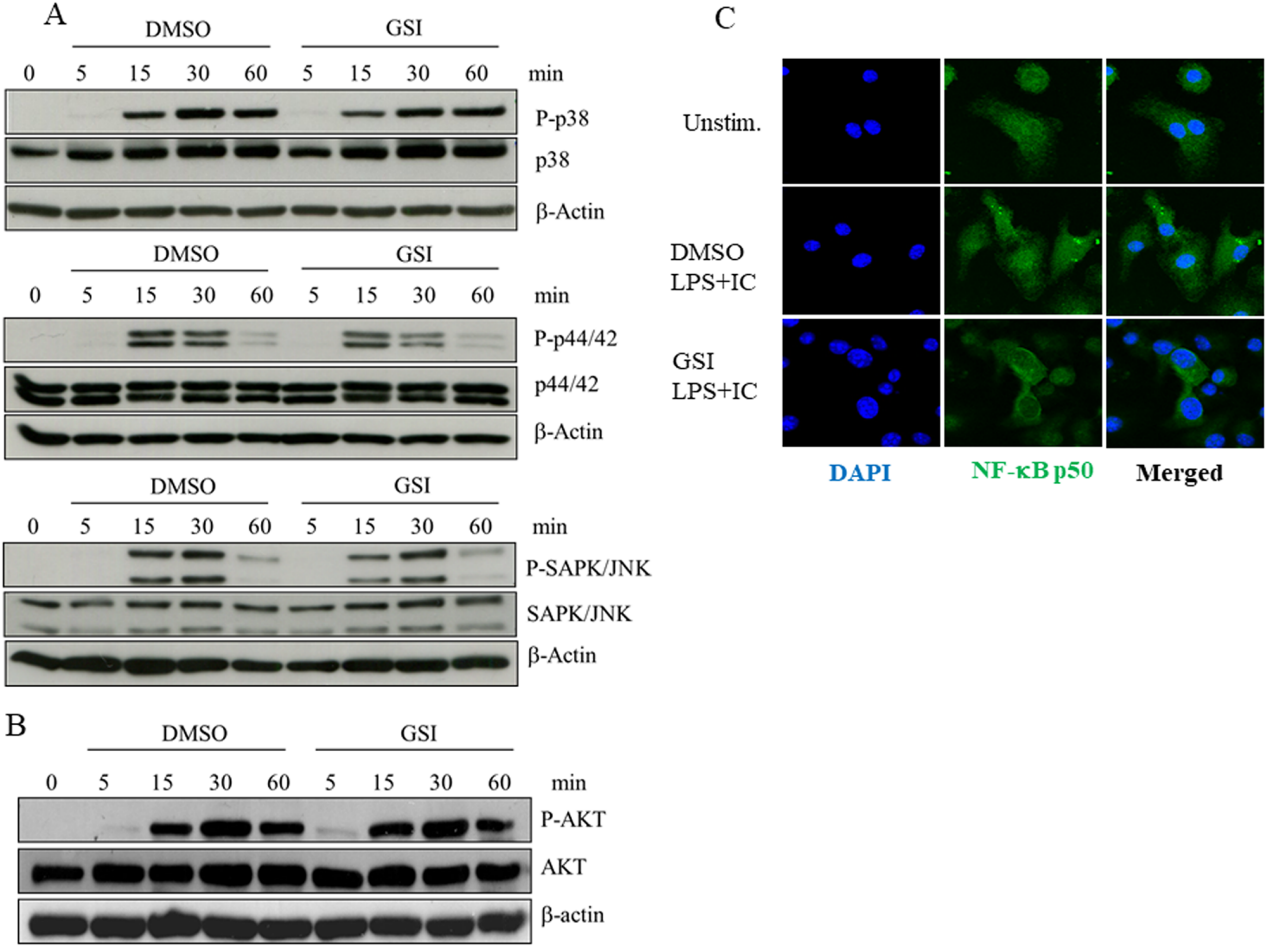
Effects of GSI treatment on MAPK, PI3K/AKT and NF-κB pathway activation in LPS/IC-activated macrophages. (A-B) IFNγ-primed BMMs were pre-treated with vehicle control DMSO or GSI (25 μM) for 30 min and activated by LPS/ICs for 5, 15, 30 and 60 min. Phospho-p38, p38 phospho-p44-42, p44-42, phospho-SAPK/JNK, SAPK/JNK, phospho-Akt, Akt and the loading control β-actin were detected by Western blotting. Representative data from 1 of 3 independent experiments are shown. (C) IFNγ-primed BMMs were activated by LPS/ICs for 4 hrs in the presence of vehicle control DMSO or GSI (25 μM). NF-κB p50 was detected by immunofluorescence staining. The arrows indicate cells with decreased or no p50 nuclear translocation (green). Representative data from 1 of 2 independent experiments are shown.

Next, we asked whether Notch signaling affects signaling downstream of TLR and FcγR by using GSI treatment. Macrophages were activated by LPS/ICs for 5, 15, 30 and 60 min in the presence of GSI or vehicle control DMSO, and the phosphorylation status of p38, Erk (p44/42), SAPK/JNK and Akt was detected. As shown in Fig 5A and 5B, the phosphorylation of p38, Erk and SAPK/JNK, which are downstream of MAPK signaling, were not affected by GSI treatment. Furthermore, the activation of PI3K also remained intact for the duration of the experiment, even when Notch signaling was inhibited. These results suggest that GSI treatment did not affect the immediate downstream signaling pathways in LPS/IC-stimulated macrophages.

**Fig 6:**
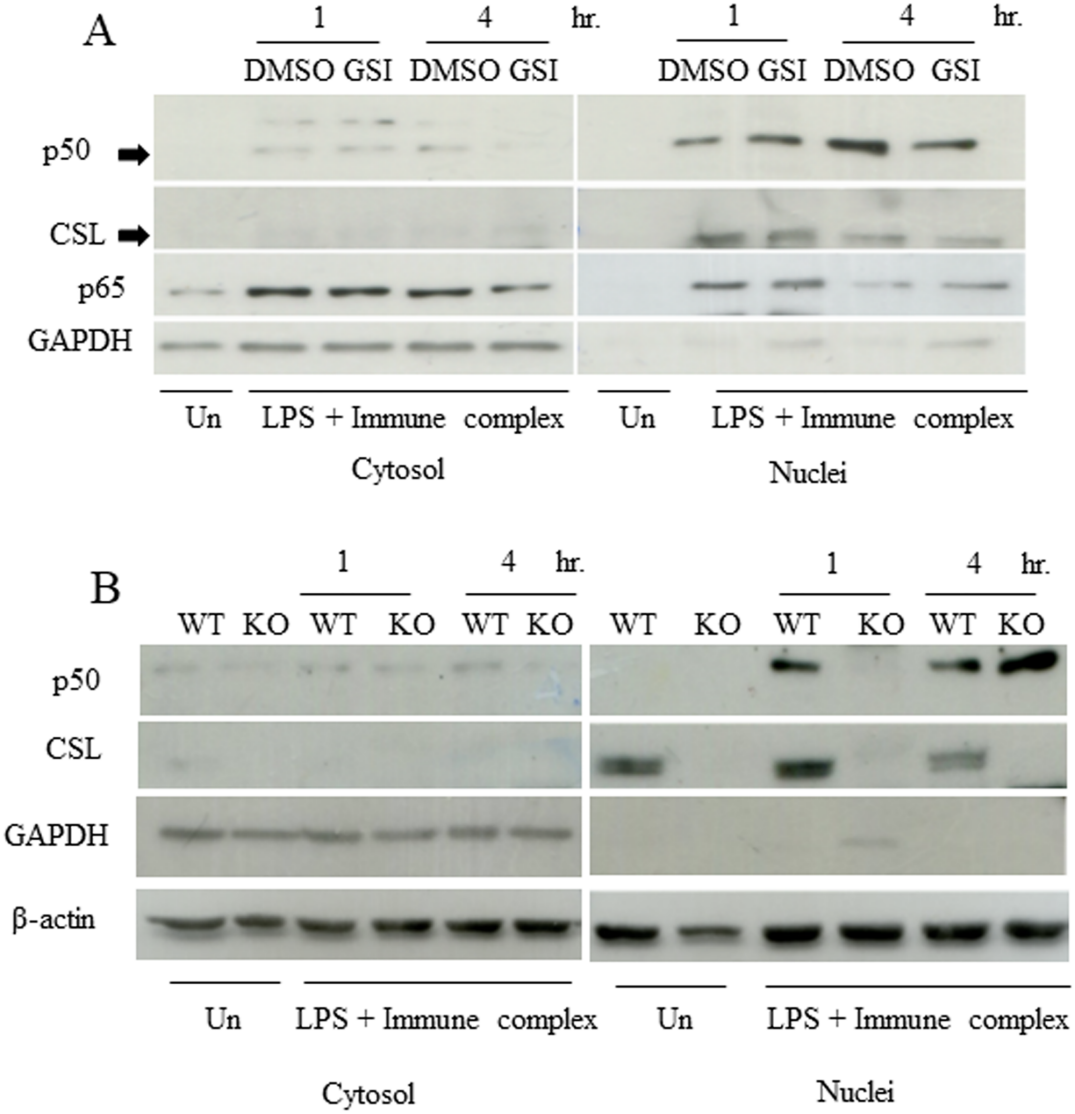
Defects in NF-κB p50 nuclei accumulation by GSI treatment or deletion of CSL/RBP-Jκ in LPS/IC-activated macrophages. (A) BMMs were pretreated with DMSO or GSI for 1 and 4 hr and the cytosolic and nuclei fractions were analyzed for NF-κB p50 by Western blotting. GAPDH and CSL/RBP-Jκ were used as cytosolic and nuclei markers, respectively. (B) BMMs from wildtype or *Rbpj* KO mice were stimulated with LPS/IC for 1 and 4 hr and the cytosolic and nuclei fractions were analyzed for NF-κB p50 as described for (A). Representative data from 1 of 2 independent experiments are shown.

When NF-κB signaling pathway activation was determined according to the nuclear localization of the p50 subunit, GSI treatment clearly suppressed the activation of p50 (Fig 5C and S2 Fig). To confirm this observation, nuclear accumulation of NF-κB p50 were examined by Western blot after separation into the cytosolic and nuclear fractions. Consistent with the results obtained from immunofluorescent staining, GSI decreased the level of nuclear NF-κB p50 at 4 hr after stimulation (Fig 6A). To examine whether similar defect occurs in *Rbpj* KO macrophages, nuclear and cytosolic fractions were prepared from wildtype or *Rbpj* KO macrophages. As shown in Fig 6B, NF-κB p50 was readily accumulated in the nuclei obtained from wild type macrophages at 1 and 4 hr after stimulation. In contrast, delay in NF-κB p50 nuclei accumulation in *Rbpj* KO macrophages was observed at 1 hr. The level of nuclei NF-κB p50 in *Rbpj* KO macrophages recovered later at 4 hr. Taken together, these data suggest that the Notch signaling pathway positively regulates NF-κB activation, at least through inhibiting the p50 subunit, and this, in turn, affects the phenotypes of LPS/IC-activated macrophages, such as IL-10 production.

### GSI treatment changes the gene expression profiles of LPS/IC-activated macrophages

**Fig 7:**
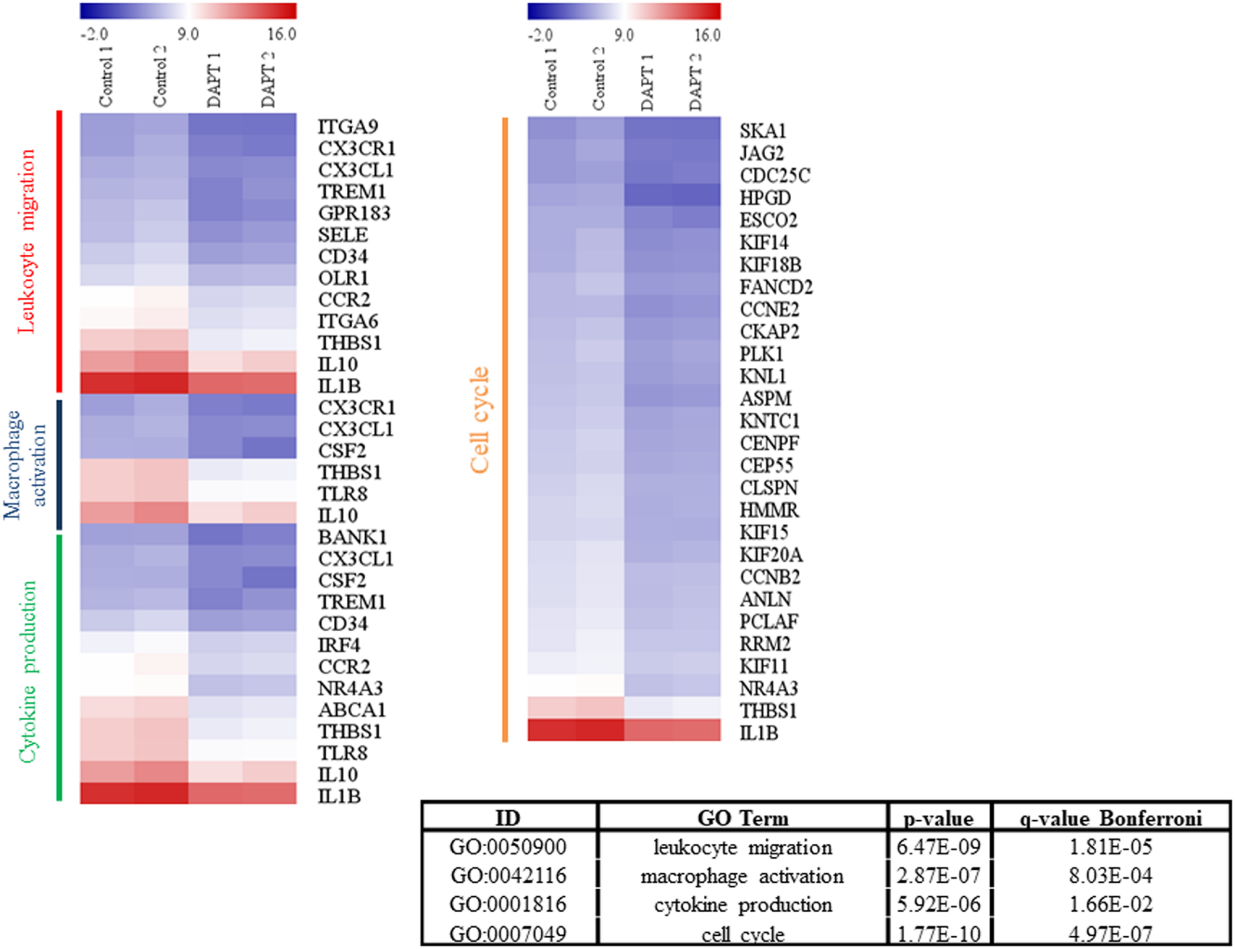
Heatmap of differentially expressed genes in LPS/IC-activated macrophages treated with DMSO *vs*. GSI. Total RNA isolated from LPS/IC-activated macrophages treated with vehicle DMSO or GSI (25 μM) was subjected to RNA-seq. Differentially expressed genes were subjected to a GO enrichment analysis. Heatmaps of genes associated with the GO terms leukocyte migration, macrophage activation, cytokine production and cell cycle are shown.

To investigate the global effect of inhibiting the activation of Notch signaling in LPS/IC-stimulated macrophages, a transcriptomic analysis by RNA-seq was performed to determine differential gene expression. When comparing BMMs stimulated with LPS/ICs in the presence of GSI and BMMs treated with vehicle control, 147 genes were found to be differentially expressed with log_2_ fold changes of greater than 1.5. More genes were downregulated in GSI-treated macrophages, suggesting that Notch signaling is positively involved in regulating gene expression (S3 and S4 Figs). The gene ontology (GO) enrichment analysis of the biological processes involving the upregulated and downregulated genes revealed that leukocyte migration, macrophage activation, cytokine production and cell cycle were significantly affected by GSI treatment in LPS/IC-stimulated macrophages (Fig 6 and S3 and S4 Figs). Interestingly, the biological processes associated with cell cycle were profoundly affected by GSI treatment in LPS/IC-stimulated macrophages (Fig 7).

**Fig 8:**
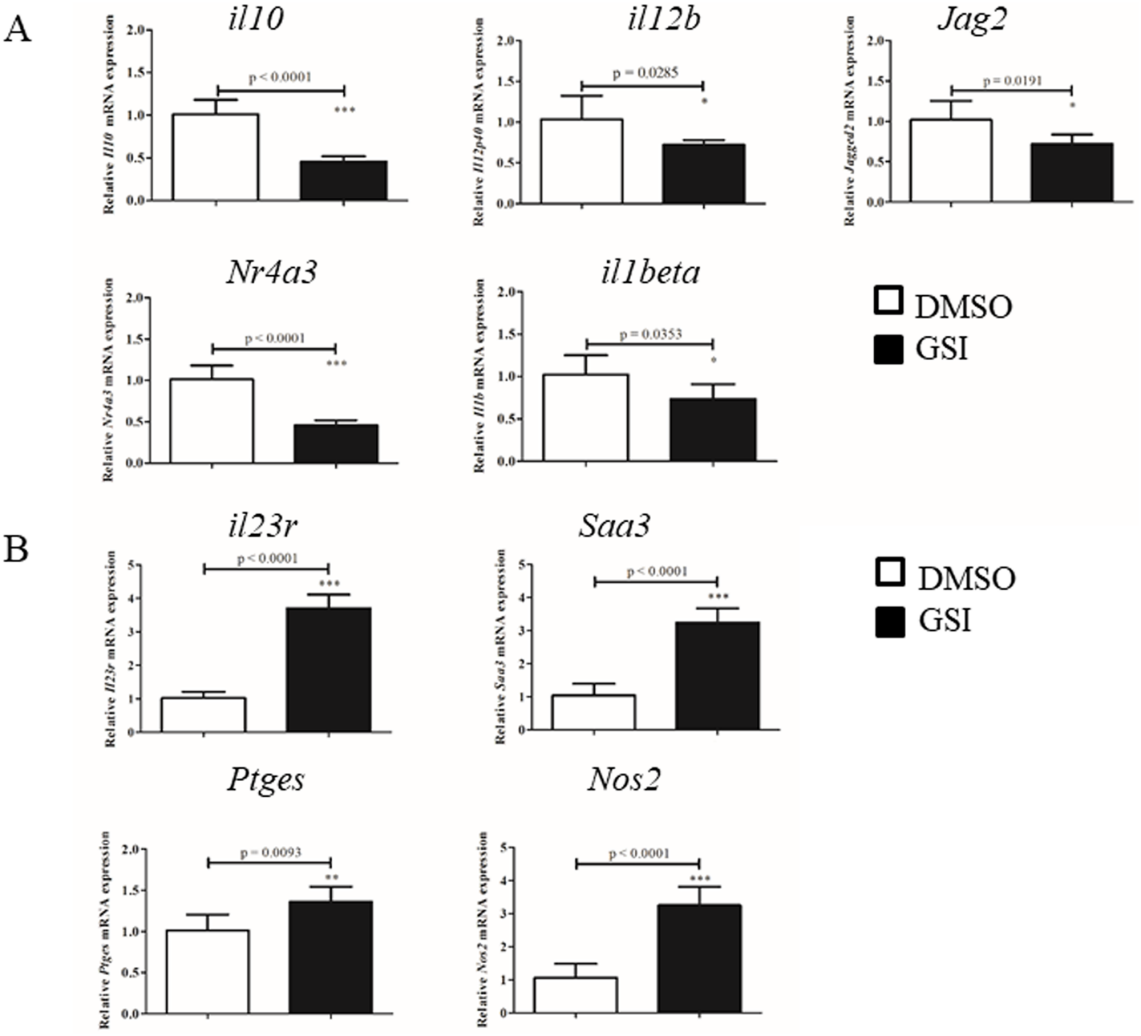
qPCR validation of differential gene expression induced by GSI treatment. Representative genes whose levels of expression were downregulated (A) or upregulated (B) by GSI treatment were validated in IFNγ-primed BMMs activated by LPS/ICs and treated with vehicle control DMSO or GSI. *, ** and *** indicate statistical significance (*p*<0.05) according to unpaired t-tests. The data represent the mean±SD of triplicate determinations from 1 of 2 representative independent experiments.

Among the genes with reduced mRNA levels, *Il10*, *Il12b*, and *Il1beta* and the Notch ligand *Jag2* and nuclear hormone receptor *Nr4a3* were validated (Fig 8A). These genes are reported to be partially regulated by the NF-κB pathway. Furthermore, the pro-inflammatory genes *Il23r*, *Saa3*, *Ptges* and *Nos2* were validated as genes upregulated by GSI treatment (Fig 8B).

**Fig 9:**
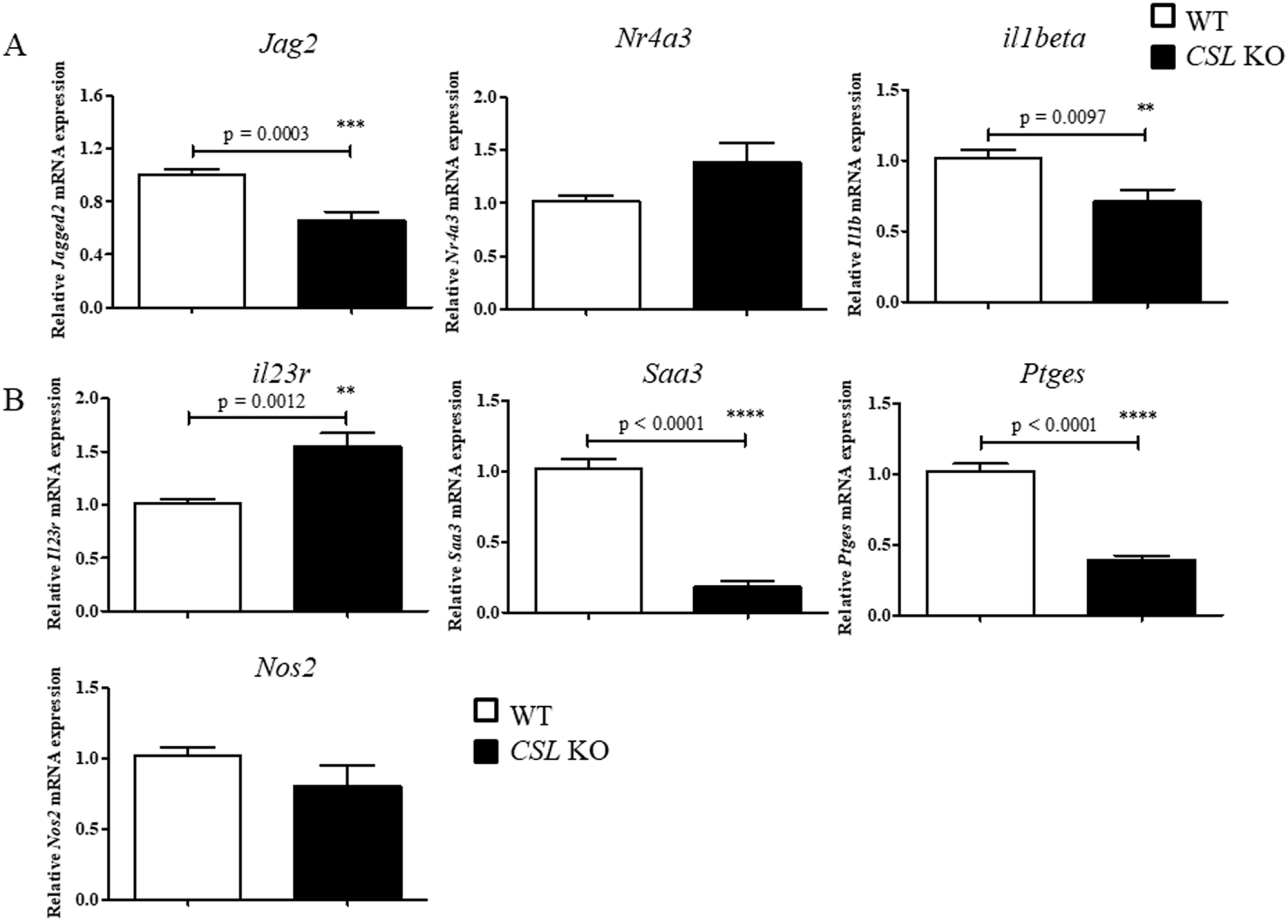
qPCR validation of differential gene expression in wildtype or *Rbpj* KO macrophages. Representative genes whose levels of expression were downregulated (A) or upregulated (B) by GSI treatment were validated in BMMs from wildtype or *Rbpj* KO mice upon activation by LPS/ICs. *, ** and *** indicate statistical significance (*p*<0.05) according to unpaired t-tests. The data represent the mean±SD of triplicate determinations from one of two representative independent experiments.

To confirm whether deletion of CSL/RBP-Jκ yields similar effect on gene expression in LPS/IC-stimulated macrophages, the same set of genes validated in Fig 8 were examined in BMMs from wildtype or *Rbpj* KO mice. As shown in Fig 9, only *Jag2* and *il1beta* mRNA showed reduced level in *Rbpj* KO macrophages, consistent with GSI treatment. In contrast, other genes showed no differences (*Nr4a3*, *il23r*, *Nos2*) or decreasing level (*Saa3*, *Ptges*). Therefore, in LPS/IC-stimulated macrophages, Notch signaling directly or indirectly suppresses inflammation via regulating the expression of inflammation-related genes. Taken together, the data indicate that Notch activation plays a complex role in regulating gene expression in LPS/IC-stimulated macrophages and may regulate inflammatory functions in this regulatory macrophage type via crosstalk with NF-κB pathways.

## Discussion

In this study, we confirmed the involvement of Notch signaling in LPS/IC-stimulated macrophages. Signaling through LPS/TLR4 is essential for Notch activation and Dll4, but not Jagged1, was identified as the main ligand that activates Notch1. An antibody or ICs alone was not sufficient to initiate Notch signaling activation as determined by the cleavage of Notch receptors. Previously, Foldi *et al* reported that the activation of Notch signaling was initiated through the downstream signaling of TLR4 in LPS-stimulated macrophages, mainly through the MAPK and NF-κB pathways and autoamplified by Jagged1 [24]. The differences in ligand usage to initiate Notch activation between LPS-stimulated *vs*. LPS/IC-stimulated macrophages may result in transduction of difference signals that affect difference cellular responses. Recent report confirmed that difference Notch ligands (Dll1 and Dll4) sends pulsatile or sustained signaling dynamics via the same Notch receptor and induces opposing cell fate during embryogenesis [34]. The surface expression of Dll4 was reduced in LPS/IC-stimulated macrophages, possibly due to the internalization of the protein after ligand/receptor ligation. In this study, Notch1 was investigated, but we found that the level of Notch2 also increased upon stimulation (Fig 2A). Therefore, multiple Notch receptors may be involved in the regulation of LPS/IC-stimulated macrophages.

The production of IL-10 in macrophages stimulated with TLR was regulated primarily by the MAPK and NF-κB signaling pathways [13]. In the presence of ICs, which trigger the signaling downstream of FcγRs, signals generated by ICs and LPS cooperate to enhance the production of IL-10 [7]. The level of IL-10 produced was affected to varying degrees when specific inhibitors (MEK1/2 MAPKs, p38 MAPK, NF-κB IκBα, PI3K and Notch) were used, indicating that each signal contributes differentially to controlling IL-10 production in macrophages activated by LPS with ICs. The limitation of this study using inhibitors is the undesirable off-target effect. Detailed characterization of the contribution of each pathway may be required to confirm this observation.

We further investigated the role of NF-κB in macrophages that were activated by LPS and ICs because NF-κB signaling was reported to control IL-10 production in macrophages stimulated with LPS [13, 17]. A previous study demonstrated the role of NF-κB in supporting MAPK signaling in LPS-induced IL-10 production in macrophages [35]. Furthermore, various DNaseI hypersensitive sites (HSs) containing NF-κB binding motifs were identified in the *Il10* locus (-55/-46), emphasizing the important role of NF-κB in the direct regulation of IL-10 production in various cell types, including macrophages [15, 17]. Among the NF-κB subunits, NF-κB1/p50 is the most abundantly found subunit in tumor-associated macrophages that plays a critical role in suppressing anti-tumor responses by decreasing IL-12 production and increasing IL-10 level [36]. In contrast to other NF-κB subunits, p50/p50 homodimer often functions as transcriptional repressor. In LPS-activated macrophages, however, p50 homodimer forms a complex with CREB-binding protein and activates transcription of *Il10* [17]. In line with this report, macrophages from *p50*^*-/-*^ mice is highly susceptible to LPS-induced sepsis [7]. These data all indicate that NF-κB p50 plays a critical role in regulating IL-10 production in macrophages. Our results added another piece of evidences that Notch signaling acts together with NF-κB p50 to regulate *Il10* expression in LPS/IC-stimulated macrophages.

How does Notch signaling cross regulate NF-κB pathway and prime macrophages for the effect of IC for optimal IL-10 production? The mechanism can be direct (physical interaction) or indirect (via other proteins). In T cell activation, Shin *et al*. showed that the intracellular domain of Notch1 in the nuclei interacts directly with NF-κB (p50) and sustains NF-κB activation to maintain T cell activation. Decreased NF-κB activation was observed in the absence of the nuclear localization of the Notch1 protein, indicating that the Notch protein regulates the activation of NF-κB, at least the p50 subunit [37]. In T cell leukemia, Notch signaling through one of its target gene, *Hes1*, sustains NF-κB activation by repressing expression of deubiquitinase CYLD. CYLD is a negative regulator of IKK complex [38]. In our study, GSI treatment and *Rbpj* KO yielded similar results of the defects in nuclear localization of NF-κB p50. Furthermore, the level of *Hes1* decreased upon GSI treatment. These data suggested that Notch/RBP-Jκ affects NF-κB p50 nuclear localization in a canonical Notch-dependent manner, possibly through Hes1 or direct association. While the effect of *Rbpj* deletion on NF-κB p50 was observed only at early time point (1 hr post stimulation), GSI treatment exhibited longer effect up to 4 hr.

The transcriptomic analysis revealed that genes involved in the cell cycle were consistently downregulated upon GSI treatment (Fig 7). Previously, it was reported that FcγR crosslinking induces cell cycle progression through the ERK pathway [39]. In addition, the transcriptomic study by Fleming *et al*. revealed that in the cluster of genes in regulatory macrophages stimulated with LPS and ICs, prostaglandin E2 (PGE2) and adenosine are associated with increased cell growth and proliferation. Accordingly, it was postulated that regulatory macrophages may contribute to homeostasis and promote cellular repair [6]. Therefore, in LPS/IC-stimulated macrophages, Notch signaling may play an important role in regulating cell growth and proliferation.

Among the genes identified to be differentially expressed by GSI treatment, the transcript levels of genes associated with pro-inflammatory functions, *Il23r*, *Saa3*, *Ptges* and *Nos2*, were increased. In contrast, only the level of *Il23r* was reduced in *Rbpj* KO macrophages, while those of *Saa3*, *Ptges* increased. These results highlighted the differences in the cellular phenotypes obtained by GSI treatment *vs*. *Rbpj* KO. The discrepancies between the two approaches may reflect the requirement for canonical vs. non-canonical Notch signaling for LPS/IC-stimulated macrophages [40]. GSI affects both pathways while *Rbpj* KO affects only the former. Furthermore, γ-secretase has various type I transmembrane substrates besides Notch receptors and some of the impacts observed by GSI treatment may be the results of inhibition of other substrates [41]. Further experiments are needed for this verification.

Macrophages function as antigen presenting cells. During antigen presentation, cytokine milieu created by macrophages can dictate helper T cell response. IL-10 producing macrophages generated by stimulation with LPS/IC decreases disease severity of EAE upon adoptive transfer, suggesting an in vivo impact on adaptive immune responses [9]. Furthermore, adoptive transfer of Notch1 deficient LPS-activated macrophages reduced IL-17 producing helper T cells in an EAE model [31]. These observations together with the findings reported here strongly suggest that modulation of Notch signaling in LPS/IC-stimulated macrophages may alter the outcome of adaptive immune responses.

We propose a model of how Notch signaling is involved in the regulation of IL-10 in LPS/IC-activated macrophages based on the results obtained in this study (see S5 Fig). In this model, Notch signaling is activated via the signaling downstream of TLR4 (signal 1), mainly by the NF-κB and Erk pathways. Notch signaling, in turn, regulates genes directly or indirectly involved in the production of IL-10 and other genes by crosstalk with the NF-κB pathway, mainly p50 subunit. Both the NF-κB and Erk pathways, in turn, activate and sustain the activation of Notch signaling, possibly through the upregulation of the Notch ligands. Therefore, the production of IL-10 in IC-activated macrophages is regulated by the NF-κB and Erk pathways in a Notch-dependent manner via TLR4 signaling. p38 and PI3K signalings via TLR4 and FcγR (signal 2) also contributes in regulating IL-10 production, possibly, in a Notch-independent manner.

In this report, we demonstrated the role that Notch signaling plays in regulating the phenotypes of LPS/IC-stimulated macrophages. In particular, Notch/Dll4 axis regulates IL-10 production in LPS/IC-stimulated macrophages by regulating NF-κB p50 nuclear localization. The results obtained in this study may help to further understand the molecular mechanisms of macrophage activation in the presence of immune complexes and may shed new light on inflammatory-related diseases, including autoimmune disorders.

## Supporting information

S1 Table List of primers used in this study. (DOCX)

S1 Fig Effect of GSI and ligand blocking antibody on *Il10* and *hes1* mRNA level in LPS/IC-activated BMDMs. (A-B) IFNγ-primed BMMs activated by LPS/ICs and treated with vehicle control DMSO or GSI for 1 or 4 hr. The relative mRNA level of *hes1* or *Il10* were measured by qPCR. ** and *** indicate statistical significance (*p*<0.05 and 0.01) according to two way ANOVA with Post Tests. The data represent the mean±SD of triplicate determinations. (C) BMMs were treated with indicated antibodies (isotype control, anti-Jagged1 antibody or anti-Dll4 antibody) during IFNγ priming. After the priming, LPS/IC were added to BMMs for 1 hr and the level of *Il10* mRNA was evaluated by qPCR. ** indicated statistical significance (*p*<0.005) according to unpaired t-tests. The data represent the mean±SD of triplicate determinations.
(TIF)

S2 Fig Images of macrophages after immunofluorescent staining of NF-κB p50 with lower magnification. BMMs from wildtype or *Rbpj* KO mice were stimulated with LPS/IC for 1 and 4 hr and the cytosolic and nuclei fractions were analyzed for NF-κB p50. Representative data from 1 of 2 independent experiments are shown.
(TIF)

S3 Fig Heat map of downregulated gene set in LPS/IC-activated BMDMs treated with GSI. (TIF)

S4 Fig Heat map of upregulated gene set in LPS/IC-activated BMDMs treated with GSI. (TIF)

S5 Fig A proposed model how Notch signaling is involved in regulating gene expression in LPS/IC-stimulated macrophages (see text for details). Red arrows indicated the links observed in this study.
(TIF)
