# Supplementary material for: Notch signaling regulates the responses of lipopolysaccharide-stimulated macrophages in the presence of immune complexes
Source: PLoS One. 2018 Jun 11;13(6):e0198609. doi: 10.1371/journal.pone.0198609 (PMC5995379; doi:10.1371/journal.pone.0198609)
Supplement: S1 Table — (DOCX) [file pone.0198609.s001.docx]

Supplementary Information

**Supplementary Table 1.** List of primers used in this study.

| Primer | Sequence (5' to 3') | | Product Size (bp) |
| --- | --- | --- | --- |
| *Il10* | Forward | TCAAACAAAGGACCAGCTGGACAACATACTGC | 421 |
|  | Reverse | CTGTCTAGGTCCTGGAGTCCAGCAGACTCAA |  |
| *Il12b* | Forward | AACCTCACCTGTGACACGCC | 309 |
|  | Reverse | CAAGTCCATGTTTCTTTGCACC |  |
| *Jag2* | Forward | TGCTGTGGAGGTGGCTGTGTCT | 151 |
|  | Reverse | TGTTTCCACCTTGACCTCGGT |  |
| *Nr4a3* | Forward | ACCCTCCAGATATGCCCTGC | 128 |
|  | Reverse | GGTCAGCTTGGTGTAGTCGG |  |
| *Il1beta* | Forward | TATACCTGTCCTGTGTAA | 121 |
|  | Reverse | TTGACTTCTATCTTGTTGA |  |
| *Il23r* | Forward | CACTGCCGACCAAGGAATCT | 119 |
|  | Reverse | GCATGAGGTTCCGAAAAGCC |  |
| *Saa3* | Forward | AACTATGATGCTGCCCGGAG | 98 |
|  | Reverse | GCTCCATGTCCCGTGAACTT |  |
| *Ptges* | Forward | AAGCCTAGCCACACCACTTC | 100 |
|  | Reverse | CCAGCAGGGCTTTCCAGTAA |  |
| *Nos2* | Forward | CCCTTCCGAAGTTTCTGGCAGCAGC | 497 |
|  | Reverse | GGCTGTCAGAGCCTCGTGGCTTTGG |  |
